# Chimeric antigen receptor T (CAR‐T) cells: Novel cell therapy for hematological malignancies

**DOI:** 10.1002/cam4.5551

**Published:** 2022-12-30

**Authors:** Samane Abbasi, Milad Asghari Totmaj, Masoumeh Abbasi, Saba Hajazimian, Pouya Goleij, Javad Behroozi, Behrouz Shademan, Alireza Isazadeh, Behzad Baradaran

**Affiliations:** ^1^ Department of Biology, Faculty of Sciences University of Guilan Rasht Iran; ^2^ Department of Clinical Immunology, Faculty of Medicine The University of Manchester Manchester UK; ^3^ Department of Microbiology, Malekan Branch Islamic Azad University Malekan Iran; ^4^ Immunology Research Center Tabriz University of Medical Sciences Tabriz Iran; ^5^ Department of Genetics, Faculty of Biology Sana Institute of Higher Education Sari Iran; ^6^ Department of Genetics and Biotechnology, School of Medicine AJA University of Medical Sciences Tehran Iran; ^7^ Department of Medical Biology, Faculty of Medicine Ege University Izmir Turkey

**Keywords:** chimeric antigen receptor T cells, hematological malignancies, immune therapy, T‐cell therapy, tumor immunology

## Abstract

Over the last decade, the emergence of several novel therapeutic approaches has changed the therapeutic perspective of human malignancies. Adoptive immunotherapy through chimeric antigen receptor T cell (CAR‐T), which includes the engineering of T cells to recognize tumor‐specific membrane antigens and, as a result, death of cancer cells, has created various clinical benefits for the treatment of several human malignancies. In particular, CAR‐T‐cell‐based immunotherapy is known as a critical approach for the treatment of patients with hematological malignancies such as acute lymphoblastic leukemia (ALL), multiple myeloma (MM), chronic lymphocytic leukemia (CLL), acute myeloid leukemia (AML), Hodgkin lymphoma (HL), and non‐Hodgkin's lymphoma (NHL). However, CAR‐T‐cell therapy of hematological malignancies is associated with various side effects. There are still extensive challenges in association with further progress of this therapeutic approach, from manufacturing and engineering issues to limitations of applications and serious toxicities. Therefore, further studies are required to enhance efficacy and minimize adverse events. In the current review, we summarize the development of CAR‐T‐cell‐based immunotherapy and current clinical antitumor applications to treat hematological malignancies. Furthermore, we will mention the current advantages, disadvantages, challenges, and therapeutic limitations of CAR‐T‐cell therapy.

## Graphical abstract

The chimeric antigen receptor T‐cells (CAR‐T) are engineered T cells that recognize tumor‐specific membrane antigens, and cause death of cancer cells. This approach has created various clinical benefits for the treatment of several human hematological malignancies.

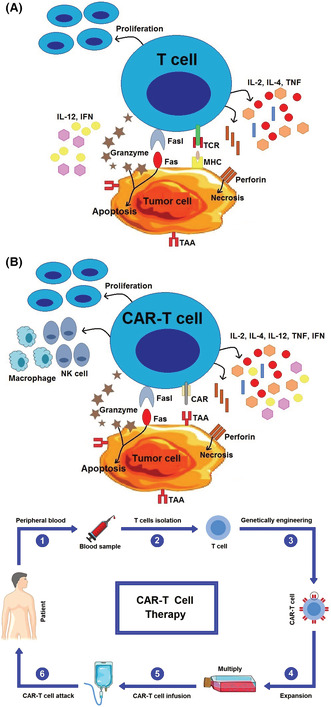

## 1 INTRODUCTION

Various therapeutic approaches have been developed during the last years for treating hematological malignancies, but these malignancies still are an important cause of cancer death worldwide.^1^, ^2^ Currently, the main treatment methods of hematological malignancies are stem cell transplantation, chemotherapy, and radiotherapy. With the increase of current knowledge about molecular genetics basis of hematological malignancies, emerging immunotherapy approaches have become a novel possibility for the treatment of these diseases. In addition, more knowledge about interaction between cancer cells and immune system cells have been a great promise for development of immunotherapy approaches.^3^, ^4^, ^5^

**FIGURE 1:**
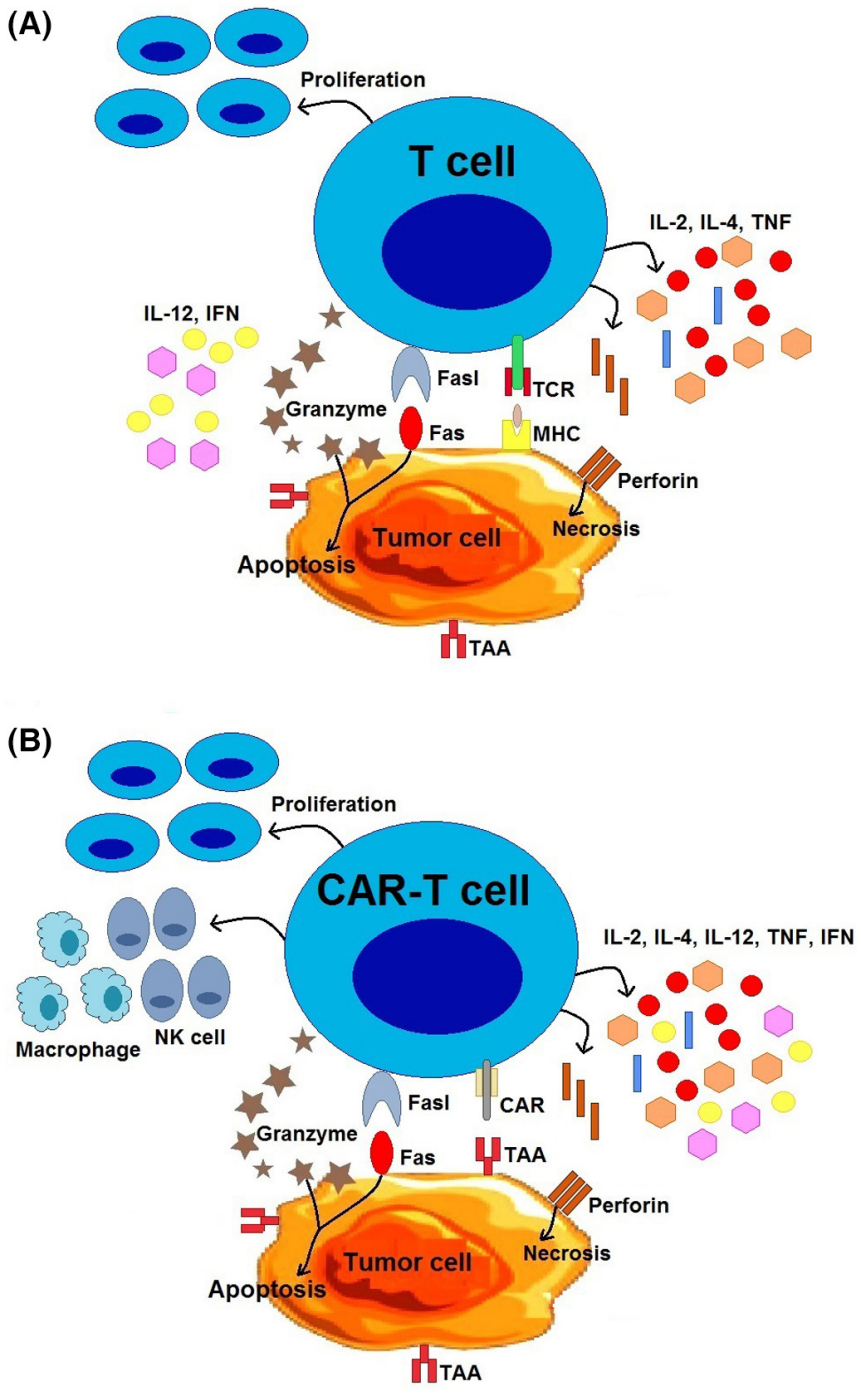
The antitumor mechanism of chimeric antigen receptors (CARs) T‐cell therapy. (A) The T‐cell receptor (TCR) recognizes intracellular and extracellular tumor‐associated antigens (TAAs) depending on presentation of MHC; but often expression of MHC downregulated by tumor cells in order to escape from killer T cells. (B) However, CAR‐T cells are able to recognize the specific TAAs in a MHC‐independent manner. Next, T cells were activated by phosphorylation of immunoreceptor tyrosine‐based activation motif (ITAM) followed by enhanced cytotoxicity, T‐cell proliferation, as well as secretion of cytokines (such as IL‐2, IL‐4, IFN‐γ, IL‐12, and TNF). Interleukin‐12 (IL‐12) recruit and reinforce functions of macrophages and NK cells. The activated CAR‐T and T cells creates cytotoxicity through production and secretion of granzyme and perforin, as well as through induction of the death receptor pathway (such as Fas/Fas‐L).

Previously, immunotherapy was deemed a potential favorable issue, but currently immunotherapy has become an applied cancer treatment approach that revolutionized the cancer therapy landscape in the past decade.^6^, ^7^ One of the most promising immunotherapeutic approaches is chimeric antigen receptor (CAR) T‐cell therapy that is highly efficient in the treatment of hematological malignancies.^8^, ^9^ This immunotherapy method prolongs the survival of patients with hematological malignancies, even if current standard therapeutic methods have failed.^10^ CAR‐T cells genetically engineered to recognizing specific tumor‐associated antigens (TAAs), and activate T cells independently of major histocompatibility complex (MHC) molecules.^11^ The antitumor mechanism of CAR‐T is summarized in Figure 1.

Immunotherapy of hematological malignant with the use of CAR‐T cells has recently provided significant progress. It has already been approved by the European Medicines Agency (EMA) and the US Food and Drug Administration (FDA) for treatment of some hematological malignancies. Besides the impressive benefits of CAR‐T‐cell therapy, recently reported serious toxicities and adverse events in some cases that have been treated with this therapeutic method. In addition, failure and relapse of CAR‐T‐cell therapy were reported in some cases.^12^, ^13^, ^14^ Therefore, further studies are needed to minimize the limitations and enhance the efficacy of this emerging immunotherapy approach.

This review study will provide the current knowledge of CAR‐T‐based immunotherapy, including current clinical application for treatment of various hematological malignancies. In addition, we will describe the advantages, disadvantages, challenges, and therapeutic limitations of this novel therapeutic approach.

## 2 CAR‐T‐CELL THERAPY

**FIGURE 2:**
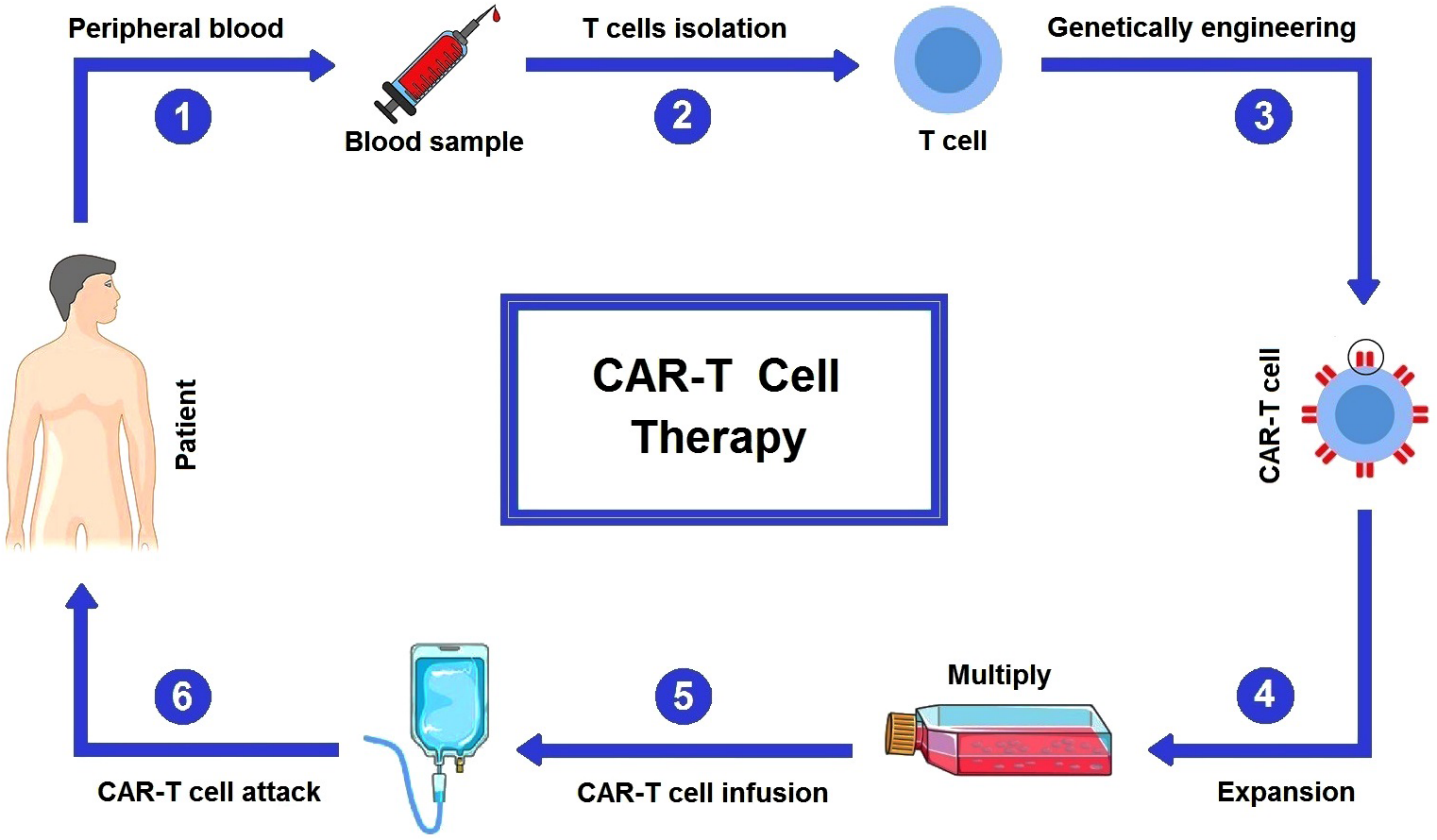
The process of CAR‐T‐cell therapy. Peripheral blood samples are taken from the patient. T cells are isolated and genetically engineered to present chimeric antigen receptors (CARs) and recognize a specific tumor associated antigen (TAAs). The obtained CAR‐T cells are expanded, and infused to the patient.

**FIGURE 3:**
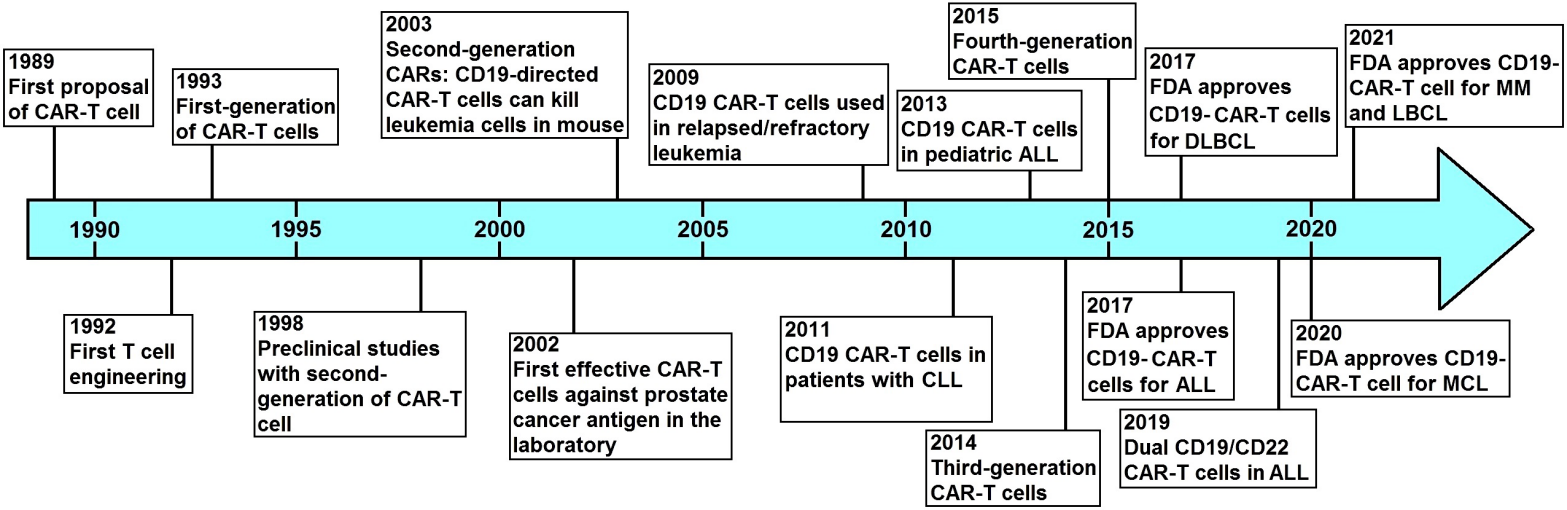
The history of CAR‐T cells progress and milestones in previous years. CAR‐T, chimeric antigen receptor‐T; ALL, acute lymphoblastic leukemia; CLL, chronic lymphocytic leukemia; DLBCL, diffuse large B‐cell lymphoma; MCL, mantle cell lymphoma; MM, multiple myeloma; LBCL, diffuse large B‐cell lymphoma.

CAR‐T‐cell therapy acts through reprogramming the immune system to combat tumor cells without any dependency on HLA presentation. The intended T cells are genetically engineered in order to presentation of monoclonal antibodies that recognize tumor‐specific antigens, and infused to the patient (Figure 2). Recognition of these cognate cancer‐specific antigens by the engineered antibodies causes to initiation of some signaling pathways in T cells that induce production of several pro‐inflammatory cytokines (IFN‐γ, TNF‐α, IL‐6, and IL‐2) and cytolysis (osmotic lysis) of cancer cells.^15^ This unique function of CAR‐T cells can help compensate for limitations of immune response mediated by T‐cell receptor (TCR), such as low affinities for antigen in T cells and MHC loss on tumor cells.^16^, ^17^ For the first time, Zelig Eshhar and Gideon Gross engineered T cells with chimeric molecule during 1989–1993 in Israel.^18^ The history of CAR‐T‐cell therapy progress and milestones is presented in Figure 3.

CARs are artificial proteins that are composed of three major components: transmembrane domain, intracellular signaling motif, and extracellular tumor‐specific antibody.^19^, ^20^ The extracellular tumor‐specific antibody is the key component in antigen targeting and incorporates a single‐chain fragment (scFv) derived from natural tumor‐specific antibodies.^21^ This component is involved in binding of CAR‐T cells to cancer cells, which subsequently stimulate activation and proliferation of T cells for production of cytokines and cytolytic degranulation.^22^ The intracellular signaling motif provides persistence, quality, and strength of T‐cell response to cancer‐specific antigens and is commonly engineered in order to increase the anticancer potency of CAR‐T cells.^21^

**FIGURE 4:**
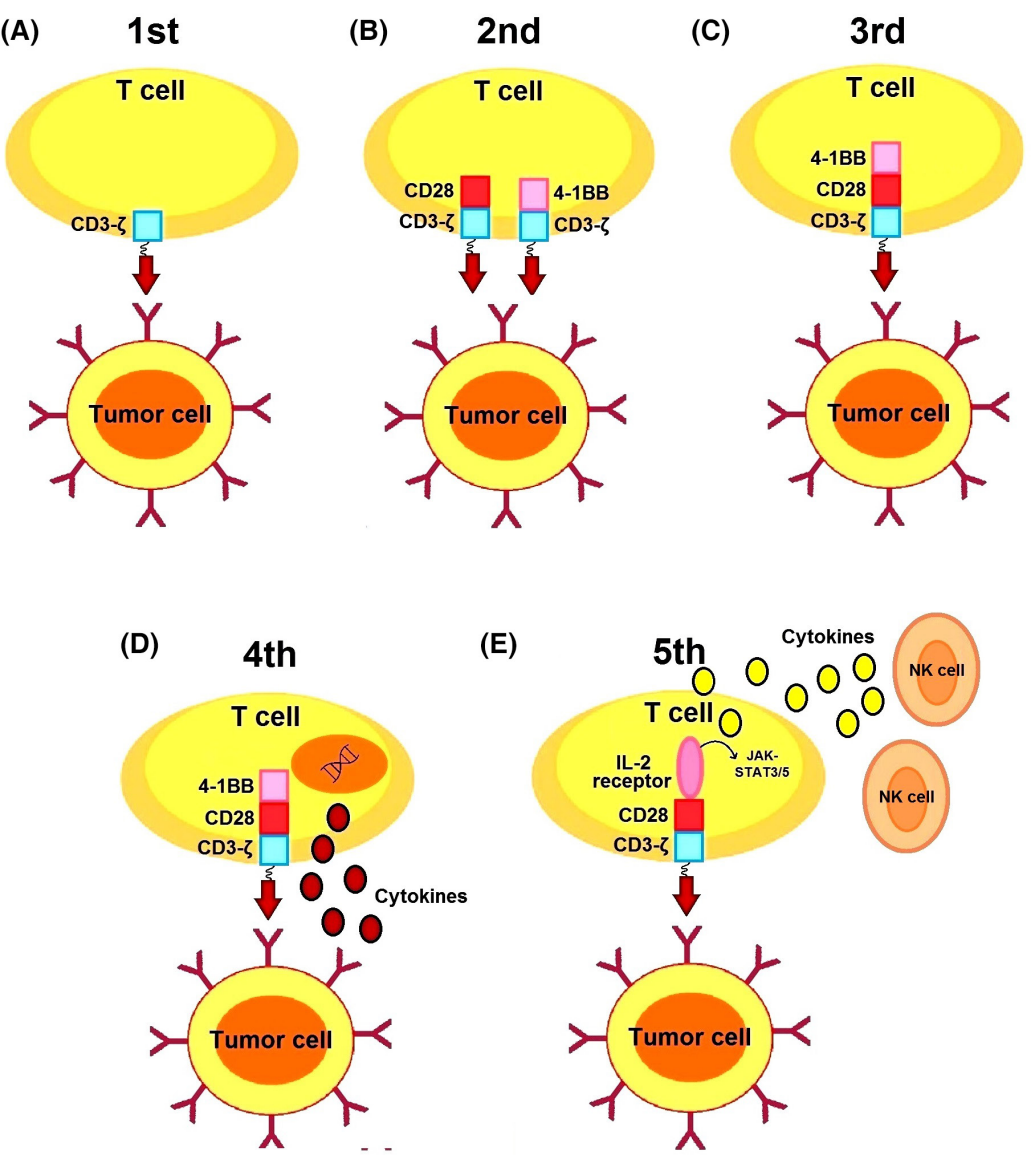
Different generations of CAR‐T cells. (A) The first generation contains only CD3ζ as an intracellular domain. (B) The second generation also consists of CD28 or 4‐1BB motifs. (C) The third generation contains both CD28 and 4‐1BB motifs. (D) The fourth generation contains IL‐12 or IL‐18 encoding genes that are tethered to the intracellular domain. (E) The fifth generation contains IL‐2 receptor and STAT3 transcription factor binding site to induce cytokine storm.

So far, five generations of CARs have been developed. In first generation, endo‐domain (intracellular signaling motif) is comprised of only CD3‐ζ chain that provides insufficient T‐cell proliferation and cytokine production.^23^ Therefore, in second generation, an intracellular co‐stimulatory domain (CD28 or 4‐1BB) has been added in order to ameliorate T‐cell proliferation and persistence.^24^, ^25^ In third generation, both CD28 and 4‐1BB have been added intracellularly in order to further increase T‐cell proliferation and persistence.^26^, ^27^ In fourth generation, various cytokines such as IL‐12 have been added to endo‐domain of the second generation of CARs, which stimulates activation of both T cells and natural killer cells against cancer cells.^28^ These stimulated natural killer cells can recruit cytokine cassettes and help increase cytotoxicity against cancer cells.^29^ This emergence prolongs the lifespan of CAR‐T cells as well as stimulates CAR‐T cells against antigen‐negative cancer cells and tumor microenvironments.^30^ Ultimately, in fifth generation, a binding site for STAT3 transcription factor and IL‐2 receptor has been added to induce cytokine storm.^28^ All five generations of CAR‐T cells are indicated in Figure 4.

Up to now, numerous approaches have been applied to increase the efficiency of CAR‐T cells' functions. Preclinical models have demonstrated that combined CD28 and 4‐1BB costimulation can lead to enhanced CAR‐T‐cell persistence, IL‐2 secretion, and cytolytic activity. The modified T cells with CD40L will lead to an increase in production and secretion of pro‐inflammatory cytokines, such as interferon‐gamma (IFNγ), tumor necrosis factor alpha (TNFα), IL‐2, and IL‐12.^31^ IL‐12 plays several critical roles in the anti‐cancer activity of CAR‐T cells through recruit and reinforce of the innate immune cells such as macrophage and NK cells, increase cytotoxic T‐cell activation, increase T helper type 1 (Th1) response, and decrease angiogenic activities.^32^, ^33^, ^34^ In this regard, T cells redirected for universal cytokine killing (TRUCK) method was developed in recent years. TRUCK can redirect CAR‐T cells through production and secretion of transgenic factors (e.g., IL‐12) in order to stimulate the immune system against cancer cells that are unrecognizable to CAR‐T cells.^35^ In addition to targeting cancer‐specific antigen, CAR‐T cells produce IFN‐γ cytokine that plays a role in antigen‐independent destruction of cancer cells through interaction with IFNγ receptors (IFNγR) that are expressed in tumor stroma.^36^

## 3 CAR‐T‐CELL THERAPY FOR HEMATOLOGICAL MALIGNANCIES

The first time in 2012, a child with acute lymphoblastic leukemia (ALL) received the CD19‐targeted CAR‐T‐cell therapy and exhibited a complete and promising response with no relapse or refractory for more than 5 years.^37^ This event provided a novel strategies of CAR‐T‐cell therapy for hematological malignancies. Afterward, several studies reported successful results with 60% to 93% remission rate as well as a minimal residual disease after CAR‐T‐cell therapy of patients with hematological malignancies.^38^, ^39^, ^40^ With rapid progress in this area, for the first time in 2017, tisagenlecleucel was approved by FDA as first CAR‐T‐cell therapy medication for treatment of under 25 years old patients with relapsed and refractory ALL.^41^, ^42^ After 2 months in the same year, the second CAR‐T‐cell therapy medication (axicabtagene ciloleucel) was approved by FDA for treatment of patients with relapsed or refractory large B‐cell lymphoma.^43^ The anti‐CD19 CAR‐T medications are the first products that received regulatory approval for treatment of patients with B‐cell ALL (B‐ALL) and B‐cell non‐Hodgkin lymphomas (NHL). Another successful example of FDA‐approved CAR‐T‐cell therapy is related to axicabtagene ciloleucel/Yescarta, (Gilead/Kite), which is used to treatment of patients with NHL.^44^, ^45^ In recent years, Gardner et al. produced CAR‐T cells that indicated 93% complete response among patients with leukemia.^38^ Another important milestone of CAR‐T products is FDA‐approved liso‐cel/Breyanzi for treatment of NHL, due to remarkable efficacy and low toxicity.^46^, ^47^ These promising results in hematological malignancies have spurred a tidal wave of clinical trials on CAR‐T‐cell therapy (Table 1).^48^, ^49^, ^50^ Due to further encouraging results in hematological malignancies, CAR‐T‐cell therapy was suggested for treatment of various solid tumors. However, the results of CAR‐T‐cell therapy in solid tumors were less efficient as compared to hematological malignancies.^51^ This can be due to limited T‐cell expansion, insufficient CAR‐T cells infiltrating and traveling to a solid tumor, poor persistence due to immunosuppressive tumor microenvironment, and low expression of target tumor‐specific antigen on the solid cancer cells.^52^, ^53^ Five FDA‐approved medications of CAR‐T cells for hematological malignancies are presented in Table 2.

**TABLE 1:** Some of the clinical trials for CAR‐T‐cell therapy of hematological malignancies.

Clinical trial	Phase	Start date	Estimated completion date	Disease	Estimated participants	Ages eligible	Target antigen	Location
NCT04599556	I/II	2020	2023	ALL	108	3–70 years (child, adult, older adult)	CD7	China
NCT01044069	I	2010	2023	ALL	93	18 ≤ years (adult, older adult)	CD19	United states
NCT02028455	I/II	2014	2036	ALL	167	1–26 years (child, adult)	CD19	United States
NCT02772198	I/II	2016	2022	ALL	300	1–50 years (child, adult)	CD19	Israel
NCT02435849	II	2015	2022	ALL	97	25 ≤ years (adult, older adult)	CD19	United States
NCT01029366	I	2010	2016	CLL	26	18 ≤ years (adult, older adult)	CD19	United States
NCT01416974	I	2011	2019	CLL	13	18 ≤ years (adult, older adult)	CD19	United States
NCT01865617	I/II	2013	2021	CLL	204	18 ≤ years (adult, older adult)	CD19	United States
NCT03331198	I/II	2017	2026	CLL	259	18 ≤ years (adult, older adult)	CD19	United States
NCT00924326	I	2009	2021	DLBCL	43	18–70 years (adult, older adult)	CD19	United States
NCT02631044	I	2016	2022	DLBCL	314	18 ≤ years (adult, older adult)	CD19	United States
NCT02348216	I/II	2015	2035	DLBCL	307	18 ≤ years (adult, older adult)	CD19	United States
NCT02445248	II	2015	2023	DLBCL	115	18 ≤ years (adult, older adult)	CD19	United States
NCT02215967	I	2014	2019	MM	30	18–73 years (adult, older adult)	BCMA	United States
NCT02658929	I	2015	2022	MM	67	18 ≤ years (adult, older adult)	BCMA	United States
NCT03958656	I	2019	2021	MM	13	18–73 years (Adult, Older Adult)	SLAM7	United States
NCT04288726	I	2020	2037	LH	18	12–75 years (child, adult, older adult)	CD30	United States
NCT04136275	I	2020	2024	LH	18	18 ≤ years (adult, older adult)	CD37	United States
NCT03904069	I	2022	2029	AML	40	12 ≤ years (child, adult, older adult)	FLT3	United States
NCT03081910	I	2017	2039	T‐ALL	42	75 ≤ years (child, adult, older adult)	CD5	United States

Abbreviations: ALL, acute lymphoblastic leukemia; CLL, chronic lymphocytic leukemia; DLBCL, diffuse large B‐cell lymphoma; MM, multiple myeloma; HL, Hodgkin lymphoma; AML, acute myeloid leukemia; T‐ALL, T‐cell acute lymphoblastic leukemia.

**TABLE 2:** The FDA‐approved CAR‐T‐cell medications for hematological malignancies.

Medication	Abecma (idecabtagene vicleucel)	Breyanzi (lisocabtagene maraleucel)	Kymriah (tisagenlecleucel)	Tecartus (brexucabtagene autoleucel)	Yescarta (axicabtagene ciloleucel)
FDA approval	Multiple myeloma: 2021	Large B‐cell lymphoma: 2021	acute lymphoblastic leukemia: 2017 Large B‐cell lymphoma: 2018	Mantle cell lymphoma: 2020	Large B‐cell lymphoma: 2017 Follicular lymphoma: 2021
CAR Construct	CD19scFv, 4‐1BB, CD3‐ζ	CD19scFv, CD28, CD3‐ζ	CD19scFv, 4‐1BB, CD3‐ζ	CD19scFv, CD28, CD3‐ζ	CD19scFv, CD28, CD3‐ζ
Vector	Lentiviral vector	Lentiviral vector	Lentiviral vector	Retroviral vector	Retroviral vector
Target antigen	Anti‐CD38 monoclonal antibody	Anti‐CD19 monoclonal antibody	Anti‐CD19 monoclonal antibody	Anti‐CD20 monoclonal antibody	Anti‐CD19 monoclonal antibody
Bridging chemotherapy	Yes: 87%	Yes: 59%	Yes: 59%	Yes: 37%	No: ‐
CAR‐T dose	450 × 10^6^ CAR‐T cells/kg	50 × 10^6^ CAR‐T cells/kg	3 × 10^8^ CAR‐T cells/kg	2 × 10^6^ CAR‐T cells/kg	2 × 10^6^ CAR‐T cells/kg
Efficacy	Overall response: 72% Complete response: 33%	Overall response: 61% Complete response: 44%	Overall response: 52% Complete response: 40%	Overall response: 85% Complete response: 59%	Overall response: 82% Complete response: 54%
Safety	Cytokine release syndrome: 84% Neurotoxicity: 18%	Cytokine release syndrome: 42% Neurotoxicity: 30%	Cytokine release syndrome: 58% Neurotoxicity: 21%	Cytokine release syndrome: 91% Neurotoxicity: 63%	Cytokine release syndrome: 93% Neurotoxicity: 64%
Side effects	Cytokine release syndrome	Cytokine release syndrome	B‐cell aplasia, off‐target activity	Cytokine release syndrome	Cytokine release syndrome

### 3.1 Acute lymphoblastic leukemia

ALL is a hematological malignancy with a high proliferation of abnormal primitive cells as well as naive cells in bone marrow. Several preclinical studies demonstrated that CAR‐T‐cell therapy is an appropriate strategy with remarkable efficacy for the treatment of ALL.^54^, ^55^ So far, several clinical trials have investigated the efficiency of anti‐CD19 CAR‐T‐cell therapy of patients with B‐ALL, which indicated promising partial remission and complete remission rates.^56^, ^57^ Two different studies from Pennsylvania and Philadelphia groups have reported that from 30 patients with ALL that received anti‐CD19 CAR‐T‐cell therapy, 27 cases (90%) indicated complete remission.^57^ In an interesting study, 57 patients with relapsed or refractory ALL were treated by CAR‐T cells, and the results indicated that 28 patients (83%) achieved complete remission.^58^ In another clinical study on 75 patients with ALL that received anti‐CD19 CAR‐T‐cell therapy reported a complete remission rate of 60%.^59^ Although anti‐CD19 CAR‐T cells is an ideal therapeutic method for ALL, often administered for patients with B‐ALL; this approach presents a limited efficacy in patients with T‐cell ALL (T‐ALL). However, a previous preclinical study on xenograft mouse models reported that anti‐CD5 CAR‐T‐cell therapy could be used effectively to treat patients with T‐ALL.^60^ Despite significant progress in this therapeutic method, several clinical trials to treatment ALL by CAR‐T‐cell therapy through targeting CD19, CD20, and CD22, as well as combination therapy by anti‐CD19 and anti‐CD20, are in progress.^61^ In a clinical trial on 27 patients with relapsed or refractory B‐ALL that received anti‐CD22 CAR‐T cells and anti‐CD19 CAR‐T cells, reported that 24 patients (89%) reached complete remission.^62^ These evidence indicates that combination and multitargeted CAR‐T‐cell therapy can be a promising therapeutic method for impressive treatment of ALL patients.

### 3.2 Chronic lymphocytic leukemia

Chronic lymphocytic leukemia (CLL) is a common subtype of leukemia that indicates poor prognosis in cases with multiple relapsed or refractory CLL.^63^ Targeting anti‐CD19 CAR‐T‐cell therapy has been introduced as an effective therapeutic method for treatment of patients with CLL. A study by Porter et al. investigated the efficiency of CAR‐T‐cell therapy through targeting CD137 and CD3zeta in patients with CLL. They reported that the number of anti‐CD137 CAR‐T cells and anti‐CD3zeta CAR‐T cells significantly expanded, and the patients were completely relieved. Moreover, they reported that the designed CARs were expressed for 6 months in bone marrow and blood of patients.^64^ In another study, Porter et al. reported that total effective rate of anti‐CD19 CAR‐T‐cell therapy was 57% among 14 patients with CLL, in which 4 patients (28%) achieved complete remission among them.^65^ In addition, combined therapy with chemotherapy and CAR‐T‐cell therapy was performed by Geyer et al. in order to treatment of eight patients with CLL.^66^ This study reported that two patients (25%) achieved complete remission for more than 28 months after treatment by infliximab chemotherapy, anti‐CD19 and anti‐CD28 CAR‐T‐cell therapy.^66^ Another study by Gauthier et al. investigated the efficiency of CAR‐T‐cell therapy along with ibrutinib in 19 patients with CLL. They reported that 83% of patients achieved complete remission. They suggested that simultaneous use of CAR‐T‐cell therapy and ibrutinib was well tolerated in patients.^67^ In addition, the possibility of concomitantly targeting CD19 and CD37 has been explored preclinically.^68^ This evidence demonstrated that CAR‐T‐cell therapy is an impressive therapeutic method for treatment of patients with CLL.

### 3.3 Acute myeloid leukemia

Acute myeloid leukemia (AML) is one of the common subtypes of leukemia in children that its main feature is uncontrolled immature myeloid cells proliferation in bone marrow. So far, CAR‐T‐cell therapy of AML has not been successful like ALL. The early efforts for CAR‐T‐cell therapy of AML were performed through targeting CD123 and CD33.^14^ In one of the first efforts, CAR‐T‐cell therapy targeting CD33 was performed in a patient with relapsed or refractory AML and reported that the tumor burden of this patient was significantly decreased in the bone marrow after anti‐CD33 CAR‐T‐cell therapy.^69^ After that, CD123 was introduced as a novel potential antigen target. However, the anti‐CD123 CAR‐T‐cell therapy indicated a low efficiency due to the relative expression of CD123 on normal cells (monocytes and endothelial cells), though it is lower than AML cells.^70^ Due to disappointing results, further preclinical studies were performed, and a large number of antigens were tried as new targets, such as Lewis‐Y (LeY) and CLEC12A.^71^, ^72^ In a phase I clinical trial study by Ritchie et al., the safety and persistence of autologous anti‐LeY CAR‐T‐cell therapy were examined in three patients with AML. They reported that one patient indicated cytogenetic remission, one patient indicated reduction of blood blasts, and one patient indicated protracted remission. However, all the three patients experienced disease progression despite the persistence of CAR‐T cells.^71^ In a recent study by Morsink et al., anti‐CLEC12A‐CD33 CAR‐T cells were applied for the treatment of a 44‐year‐old woman with AML. They reported that this female tolerated this treatment approach and achieved complete remission after 44 days of infusion.^73^ It is noteworthy that a transiently expressed mRNA anti‐CD33 CAR has been designed preclinically in order to increase the persistence of anti‐CD33 CAR‐T‐cell therapy as a potential therapeutic method for treatment of patients with AML.^74^

### 3.4 Multiple myeloma

Multiple myeloma (MM) is a neoplastic malignancy of B cell in bone marrow that its main features include monoclonal immunoglobulin production and plasma cells proliferation.^75^ In recent years, immunotherapy of MM by CAR‐T‐cell therapy has expanded. Inhibition of myeloma cells growth using CAR‐T cells against various targets (CS1, CD138, BCMA, and NKG2D) reported by preclinical studies.^76^ For the first time, a clinical trial demonstrated promising anti‐CD269 CAR‐T‐cell therapy for treatment of patients with MM.^77^ CD269, or B‐cell maturation antigen (BCMA), is a membrane antigen found on both malignant and normal plasma cells.^78^ Efficacy of BCMA CAR‐T‐cell therapy were investigated in phase I clinical trial. This study suggested that the overall response rate of this therapeutic approach was 85% among 33 patients with MM, with a 45% complete remission rate.^79^ A previous preclinical study have shown that CD138 is an effective target for the treatment of MM.^80^ In other clinical study. Heffner et al. reported a high efficiency for anti‐CD138 CAR‐T‐cell therapy in refractory MM.^81^ CD138 or syndecan‐1 is a membrane antigen found on both malignant and normal plasma cells that is an appropriate target for CAR‐T‐cell therapy.^82^ However, CD138 can also be found on the surface of epithelial cells and is not specifically found on myeloma cells. Some issues have been raised on the toxicity and specificity of this approach. Due to the absence in most tissues, BCMA is a better candidate as compared to CD138. Therefore, BCMA CAR‐T‐cell therapy is more effective and presents a great clinic outcome.^83^ The first BCMA‐directed CAR was developed less than a decade ago, showing preclinical evidence of functional targetability.^79^ Another successful experience of CAR‐T‐cell therapy is obtained by targeting CD19 in a 43‐year‐old patient with MM. A clinical study reported that five (55%) patients with MM achieved remission among nine patients after treatment by anti‐CD19 CAR‐T‐cell therapy.^84^ CD19 or B‐lymphocyte antigen expression in malignant plasma cells has been reported at lower levels as compared normal plasma cells.^85^ This data indicated that CAR‐T‐cell therapy is a promising therapeutic method for treatment of patients with MM.

### 3.5 Hodgkin lymphoma

Hodgkin lymphoma (HL) is a B‐cell malignancy, which B‐cell‐specific antigens have lost, and expression of CD30 is increased. Therefore, CD30 is an appropriate target for CAR‐T‐cell therapy of patients with HL.^14^ Despite CD30 expression on activated normal T cells as well as challenges ahead in anti‐CD30 CAR‐T‐cell therapy of HL, numerous promising results have been reported. In phase 1 clinical trial by Ramos et al., no toxicities were observed to anti‐CD30 CAR‐T‐cell therapy among seven patients with relapsed or refractory HL and reported that two patients achieved complete remission, as well as three patients achieved transient remission after treatment by anti‐CD30 CAR‐T cell.^86^ In another phase 1 clinical trial by Wang et al., patients with HL were treated by anti‐CD30 CAR‐T‐cell therapy and reported that seven patients achieved partial remission, whereas six patients remained with stable disease. They reported that all patients tolerated anti‐CD30 CAR‐T‐cell infusion without any side effects.^87^ This evidence has indicated safety, tolerability, as well as potential of anti‐CD30 CAR‐T‐cell therapy for treatment of patients with relapsed or refractory HL.

### 3.6 Non‐Hodgkin lymphoma

NHL is a group of B‐cell malignancies that includes several types of lymphomas such as DLBCL, mantle cell lymphoma (MCL), Burkitt lymphoma (BL), follicular lymphoma (FL), Li‐Fraumeni syndrome (LFS), and B‐cell lymphoblastic lymphoma (B‐LBL). Stem cell transplantation, chemotherapy, and radiotherapy are the common treatment methods for patients with NHL. However, the mortality rate from NLH has not declined. Due to remarkable success in treating relapsed or refractory lymphoma, CAR‐T‐cell therapy has recently received more attention.^14^

DLBCL is an important subtype of NHL with aggressive clinical features. In a study Jensen et al. reported that anti‐CD20 CAR‐T‐cell therapy indicated no clinical responses and toxicities in treating two patients with relapsed or refractory DLBCL.^88^ Another study by Kochenderfer et al. reported that four of seven chemoresistance patients with relapsed or refractory DLBCL achieved remission.^89^ In addition, Schuster et al. reported that 6 of 14 adult patients with DLBCL achieved remission after treatment by anti‐CTL019 CAR‐T‐cell therapy.^90^ Furthermore, Stirrups et al. used anti‐CD19 CAR‐T‐cell therapy in order to treatment of 101 patients with relapsed or refractory DLBCL. They reported that 55 cases (54%) achieved complete remission as well as 28 cases (28%) achieved partial remission.^91^

MCL is another common subtype of NHL that includes 7% of all NHL.^92^ CAR‐T‐cell therapy is an efficient therapeutic method for treatment of patients with MCL and causes complete remission in numerous of patients. In a preclinical trial, Till et al. investigated the efficiency and toxicity of anti‐CD20 CAR‐T‐cell therapy on four patients with MCL. They reported a good tolerance for this approach without any toxicity, although some transient infusion symptoms were observed in one patient. In this study, 2 patients indicated no progress for 12 and 24 months after treatment, but a partial remission occurred in 1 patient that relapsed 12 months after injection.^93^

BL is also a common subtype of NHL that a high proportion of patients indicate poor prognosis after chemotherapy. In a clinical study by Du et al., anti‐CD19, anti‐CD20, and anti‐CD22 CAR‐T‐cell therapy were applied for treatment of an 8‐year‐old boy with BL. They observed no obvious response after treatment by anti‐CD19 CAR‐T‐cell therapy. However, by anti‐CD22 CAR‐T‐cell therapy, the child experienced partial remission, but the disease relapsed quickly, unfortunately. Finally, an encouraging result was obtained after treatment with anti‐CD20 CAR‐T cell, and the patient achieved remission.^94^

In addition, several studies also reported the high efficiency of CAR‐T‐cell therapy for treatment of other NHL. A recent phase IIa study Schuster et al. investigated the efficiency of anti‐CTL019 CAR‐T‐cell therapy in 14 patients with follicular lymphoma (FL). They reported a disease progression after treatment by anti‐CTL019 CAR‐T‐cell therapy within 2 years.^90^ In another study, Neelapu et al. treated 66 patients with aggressive and refractory NHL by FMC‐63, a single‐chain antibody that recognizes CD19 on cancer cells. They reported 52% complete effective rate as well as 79% total effective rate.^95^ Moreover, Chen et al. evaluated efficiency of anti‐CD19 and anti‐CD22 CAR‐T‐cell therapy in a patient with relapsed or refractory acute B‐LBL. They reported a complete tumor remission in the studied patient.^96^

## 4 DISADVANTAGES AND CHALLENGES

### 4.1 Cytokine release syndrome

Cytokine release syndrome (CRS), a systemic immune inflammation, rapidly produces and secretes inflammatory cytokines after injection of CAR‐T cells to patients. CRS is known as a most important side effect of CAR‐T‐cell therapy, which commonly causes several signs such as hypoxia, fever, hypotension, and neurological alterations.^97^ The diagnostic criteria for severe CRS can be investigated by systemic analysis of serum cytokines as well as clinical analysis 21 days after injection of CAR‐T cell. Moreover, serum levels of C‐reactive protein (CRP) is a dependable factor in order to investigate severity of CRS and is a disease management way in clinical centers that presents CAR‐T‐cell therapy.^98^ Tocilizumab is a humanized monoclonal antibody against IL‐6 receptor, which was approved by FDA for treatment of CRS. After taking tocilizumab, CRS subsides rapidly and does not affect the efficiency of CAR‐T‐cell therapy.^99^ A study by Caimi et al. reported that use of prophylactic tocilizumab followed by anti‐CD19 CAR‐T‐cell therapy cause reduce of incidence and severity of CRS.^100^ In another study, Jiang et al. reported that severe CRS after CAR‐T‐cell therapy could cause disseminated intravascular coagulation (DIC). They suggested that corticosteroids and immunosuppressive agents could be used to prevent CRS‐related coagulation and appropriate management of CAR‐T treatment.^101^

### 4.2 Neurotoxicity

Neurotoxicity is one of the main side effects of CAR‐T‐cell therapy that is associated with numerous symptoms such as confusion, delirium, seizures, and mild headaches, visual hallucination, acute encephalopathy, and cerebral edema.^102^, ^103^ The onset of neurotoxicity is less than CRS and usually occurs after CRS onset and a few days after CAR‐T‐cell therapy. Pathogenesis of neurotoxicity is unclear and may be correlated with T‐cell trafficking or cytokines diffusion in the brain.^40^, ^104^ Neurotoxicity is usually solved within a few days and is uncommon after a perfect treatment.^40^ Strategies to deal with the CAR‐T‐cell‐associated neurotoxicity are aimed to reduction of inflammatory response. Siltuximab is a IL‐6 antagonist monoclonal antibody that prevents translocation of IL‐6 from blood–brain barrier (BBB) and plays an important role in managing neurotoxicity.^102^ Antiepileptic agents or levetiracetam are other drugs prevention for severe neurotoxicity as well as seizures prophylaxis.^105^ However, further studies are required to optimize the management of neurotoxicity after CAR‐T‐cell therapy and identify underlying molecular mechanisms and risk factors of neurotoxicity.

### 4.3 On‐target/off‐tumor toxicity

On‐target off‐tumor is toxicity specific to CAR‐T‐cell therapy resulting from a direct attack on normal tissues.^106^ This event may occur in the form of manageable depletion as B‐cell aplasia or severe toxicity, which observed in various organ systems such as hematologic, pulmonary, and gastrointestinal.^107^ B‐cell aplasia, absence, and elimination of B cells, commonly occur after anti‐CD19 or anti‐CD22 CAR‐T‐cell therapy cause various types of infectious diseases.^108^, ^109^ Persistence and efficacy of CAR‐T‐cell therapy can be investigated by B‐cell aplasia rate. B‐cell aplasia also can be used for prediction of disease relapse.^109^ To overcome this toxicity, several novel CARs are designed that are able to distinguish malignant and healthy cells. These CAR constructs include masked CARs, inhibitory CAR (iCAR), universal CARs (UniCARs), and Logic‐Gated CAR‐T Cells.^110^ One of the important key to success of CAR‐T‐cell therapy is detection of a specific antigen that expressed on tumor cells surface, but not expressed on normal cells. CD19 is one of the promising target that expressed on surface of B‐cell malignancies.^51^ However, clinical studies have reported that relapse rate is approximately 30% after anti‐CD19 CAR‐T‐cell therapy, which may be due to low persistence of CAR‐T cells, antigen escape, antigen loss, and antigen downregulation. In addition, other targeted antigens may lead to on‐target/off‐tumor toxicities that is unacceptable or even fatal.^59^, ^111^ Therefore, combination of multi‐antigen targets is a potential strategy to increase effectiveness of CAR‐T‐cell therapy. In this regard, Boolean logic gates system (AND, OR, and NOT) has been introduced to improving multi‐antigen targeted CAR‐T‐cell therapy, reduce on‐target/off‐tumor toxicities, and prevent tumor antigen escape.^112^, ^113^

## 5 THERAPEUTIC LIMITATIONS

CAR‐T‐cell therapy has become an encouraging therapeutic method for treatment of various hematological malignancies, but there are still several limitations for broadly application of this therapeutic method. The availability and cost are the first important factors that limits application of CAR‐T‐cell therapy. The modified CAR‐T cells are highly personalized and produced from immune cells isolated from the patient. In contrast to the other immunotherapeutic approaches (such as inhibition of immune checkpoints), CAR‐T cells cannot be mass‐produced and not universal. These factors cause to increase costs of therapy and decrease number of equipment and facilities that required for an appropriate therapy. Various advanced instrument and technologies (such as gene‐editing tools and viral vectors) are required for genetic modification of T cells that may not be available in smaller therapeutic centers and laboratories. Moreover, a highly sterile and controlled fully equipped environment as well as continuous monitoring is essential in order to avoid infections in patients that received CAR‐T‐cell therapy.^114^, ^115^

In addition, possibility of resistance to CAR‐T‐cell therapy is one of the important limitation that can be occur in response to prolonged exposure to genetically engineered CAR‐T cells. Resistance to CAR‐T‐cell therapy especially is observed in ALL patients with negative CD19 expression.^116^

The numerous barriers and several complex mechanisms have characterized that cause to transient improvement as well as decrease efficiency of CAR‐T‐cell therapy. One of the main cause of treatment failures by CAR‐T‐cell therapy is limited persistence or insufficient expansion of genetically engineered T cells in body of the patient. Another important cause of therapy failure is lower or loss of antigen that can occur in some patients. Relapse of malignancy in some patients cause that malignant tumor cells no longer express the TAAs targeted by the first modified CAR‐T cells.^117^ The majority of CAR targets are TAAs that are upregulated on surface of cancer cells.^118^, ^119^ The risk of on‐target off‐tumor toxicity is associated with overexpression of TAAs on surface of nonmalignant cells. Low expression of TAAs on nonmalignant cells minimize the risk of on‐target off‐tumor toxicity.^120^ The combinatorial antigen is a most common strategy for increase specificity of CAR‐T‐cell therapy. This method increases ability of CAR‐T cells to discriminate between target and off‐target cells.^121^

**FIGURE 5:**
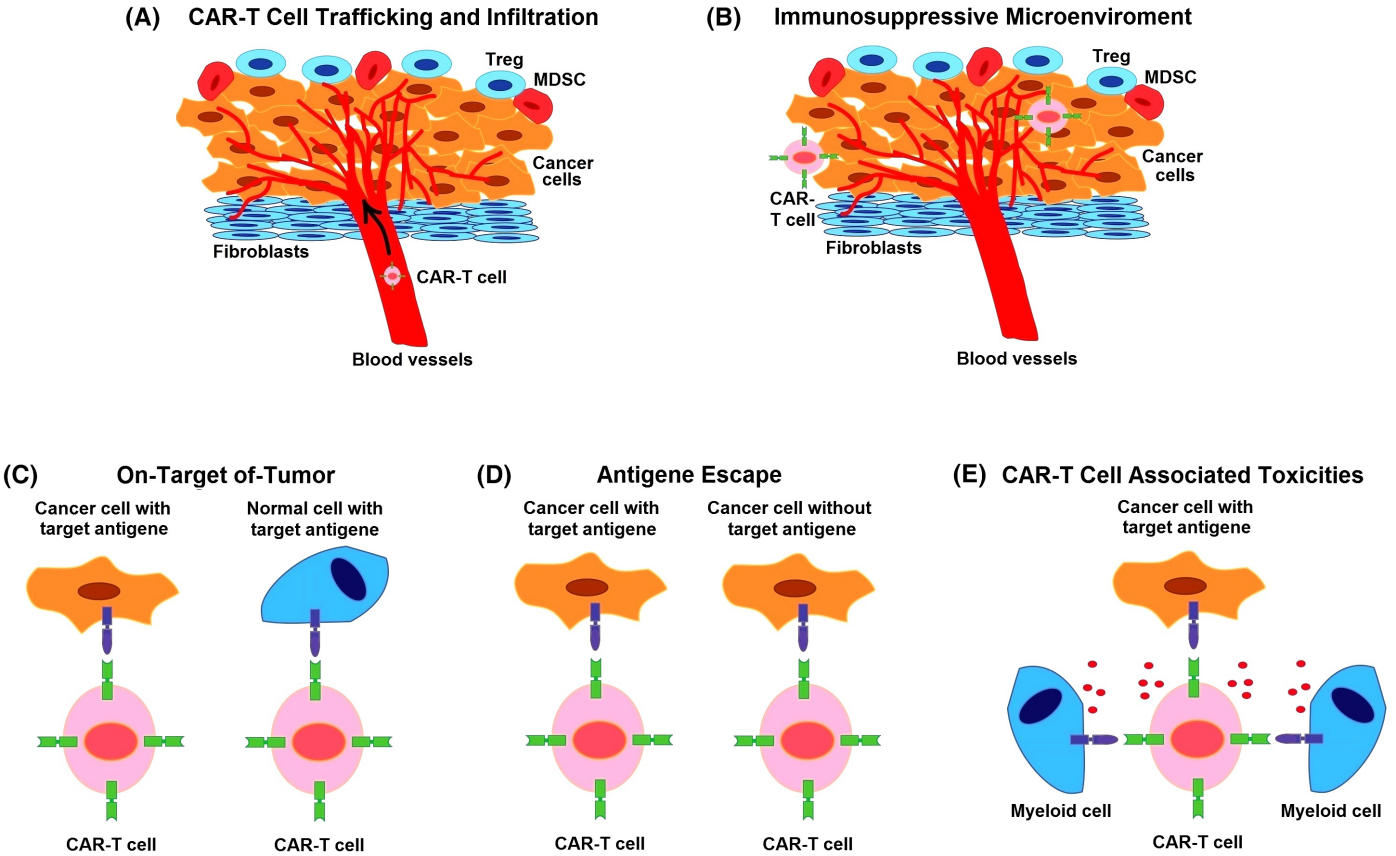
Limitations in use of CAR‐T‐cell therapy. (A) Immunosuppressive tumor microenvironment or engineering CARs cells to overcome to immunosuppressive factors. (B) CAR‐T trafficking and infiltration of tumors or engineering CARs that increase penetration from physical barriers. (C) On‐target/off‐tumor effects or binding to target antigen on cancer cells that also expressed on normal cells. (D) Antigen escape or design of a CARs that able to target multiple antigens. (E) CAR‐T‐cell‐associated toxicities or alteration of CARs structure to ameliorate toxicity.

The other main technically limitations of CARs therapy are include: immunosuppressive tumor microenvironment (design of a CARs that able to overcome immunosuppressive factors such as immune checkpoints), CAR‐T trafficking, and infiltration of tumors (design of a CARs that increase penetration from physical barriers), On‐target/off‐tumor effects (binding to target antigen on cancer cells that also expressed on normal cells), CAR‐T‐cell‐associated toxicities (alteration of CARs structure to ameliorate of toxicity), and antigen escape (design of a CARs that able to target multiple antigens),^117^ which are current challenges in extensive use of this approach (Figure 5).

## 6 FUTURE DIRECTION

In recent years, CAR‐T‐cell therapy has provided enormous development in treatment of hematological malignancies. However, there are still numerous challenges and limitations that need to be addressed. The main problems in in this therapeutic approach are include increase of durability and effectiveness of CAR‐T cells in body of patients as well as decrease the side effects after CAR‐T‐cell therapy.^106^

The durability and effectiveness of CAR‐T‐cell therapy can be improved through use of oncolytic viruses' carrier of chemokine encoding genes to more recruit CAR‐T cells. Previous experimental studies demonstrated that oncolytic viruses are able to increase duration of exposure to CAR‐T cells as well as directly attacks malignant cells, which may have great potential to increase permanence and efficiency of CAR‐T‐cell therapy for treatment of human hematological malignancies.^122^, ^123^

The side effects after CAR‐T‐cell therapy also can be partially resolved through application of corticosteroids and tocilizumab as the main therapeutic drugs.^124^ Moreover, eliminate of CAR‐T cells by several strategies after a period of improvement can reduce CAR‐T‐cell‐associated toxicities.^124^ Constructs that allow switching the CAR expression on and off are currently in preclinical development and if successful would provide better control of CAR‐T‐related toxicity.^125^ Preclinical evidence demonstrated that the main strategies include use of anti‐CD19 CAR‐T‐cell‐mediated B cell that eliminate CAR‐T cells by B cells as well as use of suicide gene system such as induced caspase 9 (iCas9) dimerization that eliminate CAR‐T cells by cell death.^126^, ^127^ These strategies may avoid the side effects after CAR‐T‐cell therapy, and provide a novel perspective for future directions.

Furthermore, the problem of access to patients' autologous T cells and high cost of therapy can be addressed through development of universal CARs. Application of clustered regularly interspaced short palindromic repeats (CRISPR) in order to modification of allogeneic genes can offers high potential to production of universal CAR‐T‐cell therapy for treatment of hematological malignancies.^128^, ^129^

## 7 CONCLUSION

CAR‐T‐cell therapy has provided potential for treatment of numerous hematological malignancies such as AML, ALL, CLL, MM, HL, and NHL. The main aim of this therapy is screen out tumor‐specific target antigens and design of CAR‐T cells to injection to patients against tumor cells. This strategy has been applied relatively successful in clinical treatment of hematological malignancies for, and have gained headway in this field. However, there are still several disadvantages, such as neurotoxicity, CRS, and off‐tumor toxicity that decrease efficiency as well as side effects of CAR‐T‐cell therapy. Therefore, further studies are required to identification of underlying molecular mechanisms and overcome these deficiencies.

## AUTHOR CONTRIBUTIONS

Samane Abbasi, Milad Asghari Totmaj, Masoumeh Abbasi, Saba Hajazimian, and Pouya Goleij wrote the manuscript. Javad Behroozi and Alireza Isazadeh prepared figures for the manuscript. Alireza Isazadeh, and Javad Behroozi, and Behzad Baradaran edited and provided comments to improve the manuscript. Behzad Baradaran performed significant studies in the subject area of this manuscript.

## CONFLICT OF INTEREST

All authors declare no conflict of interest.

## ETHICAL STATEMENT

Being a review article, ethical committee approval was not required.

## Data Availability

All the information provided in the article are available.
